# Tracing Links Between Early Auditory Information Processing and Negative Symptoms in Schizophrenia: An ERP Study

**DOI:** 10.3389/fpsyt.2021.790745

**Published:** 2021-12-20

**Authors:** Giulia M. Giordano, Francesco Brando, Andrea Perrottelli, Giorgio Di Lorenzo, Alberto Siracusano, Luigi Giuliani, Pasquale Pezzella, Mario Altamura, Antonello Bellomo, Giammarco Cascino, Antonio Del Casale, Palmiero Monteleone, Maurizio Pompili, Silvana Galderisi, Mario Maj, The Italian Network for Research on Psychoses, Eleonora Merlotti

**Affiliations:** ^1^Department of Psychiatry, University of Campania “Luigi Vanvitelli”, Naples, Italy; ^2^Department of Systems Medicine, University of Rome Tor Vergata, Rome, Italy; ^3^Department of Clinical and Experimental Medicine, Psychiatry Unit, University of Foggia, Foggia, Italy; ^4^Department of Medicine, Surgery and Dentistry “Scuola Medica Salernitana”, Section of Neurosciences, University of Salerno, Salerno, Italy; ^5^Department of Neurosciences, Mental Health and Sensory Organs, S. Andrea Hospital, University of Rome “La Sapienza”, Rome, Italy

**Keywords:** schizophrenia, negative symptoms, EEG, ERP, N100

## Abstract

**Background:** Negative symptoms represent a heterogeneous dimension with a strong impact on functioning of subjects with schizophrenia (SCZ). Five constructs are included in this dimension: anhedonia, asociality, avolition, blunted affect, and alogia. Factor analyses revealed that these symptoms cluster in two domains: experiential domain (avolition, asociality, and anhedonia) and the expressive deficit (alogia and blunted affect), that might be linked to different neurobiological alterations. Few studies investigated associations between N100, an electrophysiological index of early sensory processing, and negative symptoms, reporting controversial results. However, none of these studies investigated electrophysiological correlates of the two negative symptom domains.

**Objectives:** The aim of our study was to evaluate, within the multicenter study of the Italian Network for Research on Psychoses, the relationships between N100 and negative symptom domains in SCZ.

**Methods:** Auditory N100 was analyzed in 114 chronic stabilized SCZ and 63 healthy controls (HCs). Negative symptoms were assessed with the Brief Negative Symptom Scale (BNSS). Repeated measures ANOVA and correlation analyses were performed to evaluate differences between SCZ and HCs and association of N100 features with negative symptoms.

**Results:** Our findings demonstrated a significant N100 amplitude reduction in SCZ compared with HCs. In SCZ, N100 amplitude for standard stimuli was associated with negative symptoms, in particular with the expressive deficit domain. Within the expressive deficit, blunted affect and alogia had the same pattern of correlation with N100.

**Conclusion:** Our findings revealed an association between expressive deficit and N100, suggesting that these negative symptoms might be related to deficits in early auditory processing in SCZ.

## Introduction

Negative symptoms represent an unmet therapeutic need in the care of subjects with schizophrenia (SCZ) (1, 2). Indeed, these symptoms do not respond satisfactorily to current available treatments and are regarded as one of the main determinants of the poor outcome of SCZ (1–6). According to the present conceptualization, negative symptoms are described as five individual symptoms: avolition (reduced interest and motivation for goal-directed activities), asociality (diminished social drive or interest and desire for affiliation), anhedonia (reduced ability to experience or anticipate pleasure), blunted affect (reduced intensity and range of emotional expression), and alogia (reduced spontaneous speech and loss of conversational fluency) (2, 7–10). Different factor analytic studies demonstrated the existence of two negative symptom domains, which are named as experiential domain, including anhedonia, avolition, and asociality, and the expressive deficit domain, including blunted affect and alogia (2, 8, 10–14). Clustering into two domains is also supported by studies that showed how these domains are associated with different behavioral and neurobiological alterations (8, 10, 15, 16). The experiential domain is associated with abnormalities in different aspects of the motivational processes, which might be related to the motivational value system (research domain criteria-RDoC-positive valence system) (17, 18) or to the salience system (8, 19). The former refers to motivational aspects such as reward prediction, value encoding, action outcome contingency learning, and the integration of goal-directed behavior and experienced value (8, 10, 16, 20–45). On the other side, the salience system refers to motivational aspects related to orientation toward salient stimuli (aversive or rewarding stimuli), cognitive activation, and general motivation (8, 46–48). Another hypothesis, which has not been entirely supported by previous studies (8), poses at the bases of the experiential domain deficits in the executive control of behavior (16, 49–53).

The pathophysiology of the expressive deficit domain has been less investigated, in comparison to the experiential domain (8). Symptoms that belong to the expressive deficit have been found to relate to deficits in neurocognitive and social cognition abilities and to neurological soft signs, suggesting that these symptoms are probably subtended by a diffuse neurodevelopmental disconnectivity (8, 54, 55). In particular, it is possible that the expressive deficit domain is related to limited availability of cognitive resources. According to this hypothesis, alogia might depend on deficits in semantic memory organization and verbal fluency. Furthermore, it has been suggested that in “high-load” situations (e.g., social situations) subjects might allocate less cognitive resources to speech production due to the high cognitive demands required from the surrounding environment (15). Another hypothesis has indicated emotion expression and emotion perception deficits as possible candidate mechanisms that subtend this domain and in particular blunted affect (15, 56, 57).

Electrophysiology (EEG), which is a non-invasive and inexpensive technique with a high temporal resolution, represents a valid method to identify abnormalities of cortical brain functions and to investigate the neurophysiological bases of different psychopathological aspects, such as negative symptoms (58–61). Specifically, the analysis of event-related potentials (ERPs) represents an objective tool to study mental processes, due to its high temporal resolution in capturing responses to internal and external events (62, 63). However, so far, findings regarding associations between ERPs and negative symptoms are scattered/scarce and often inconsistent. Three studies investigated abnormalities of reward anticipation and evaluation processes (assessed using the stimulus preceding negativity-SPN, P300, and N200) and their eventual association with negative symptoms (64–66). Wynn et al. (66) found that the SPN was associated with trait anhedonia and with the total negative symptoms score. P300 (64) and N200 (65) amplitude did not correlate with the two negative symptom domains, while the P300 amplitude was found to be associated with social anhedonia (64). However, other studies found that these ERP indices correlated also with other psychopathological aspects, for instance P300 was associated also to positive and disorganized dimensions. The inconsistence about previous findings might be due to different factors, such as the heterogeneity of negative symptoms, the improper conceptualization of these symptoms, the use of assessment instruments often not in line with the current conceptualization of negative symptoms and the small sample sizes of the studies.

Another ERP that has been extensively studied in SCZ is the N100, which is thought to measure early perceptual processing. N100 is one of the largest auditory and visually evoked ERP and can be visualized as a negative deflection peaking between 80 and 120 ms after the stimulus onset (67). The N100 has gained attention due to the fact that its alterations (a reduction in N100 amplitude and delayed latency of its peak) represent well-replicated findings in SCZ, since the early phases of the disorder (67–78). Furthermore, aberrations of N100 in schizophrenia include also deficits in N100 gating ratio probably due to decreased N100 amplitude to initial stimulus, whereas the N100 amplitudes to the repeated stimulus did not systematically vary between patients and controls (79). Previous findings demonstrated that abnormalities in sensory gating and decreased N100 amplitude might be associated with deficits in processing of auditory salience, auditory verbal hallucinations (80–82), antipsychotic intake (67), and attention deficits (83, 84). Subjects with primary and persistent negative symptoms demonstrated a reduction of N100 but not of other ERP components such as P300 (69, 85), suggesting a link between early information processing and primary negative symptoms.

Alterations in N100, reflecting deficits in gating and early sensory processing, are consistently found in SCZ, and contribute to poor outcome (86–88). Indeed, some “cascade” models have hypothesized that impairment in early visual and auditory processing might contribute to deterioration of higher-level processing, such as social cognition. These deficits might be related to negative symptoms and might contribute to impairment in functioning (86–88).

Therefore, it seems to be of great interest the investigation of eventual associations between N100 impairment and negative symptoms. However, also in this case findings are inconsistent. In particular, two studies investigated abnormalities in ERPs in subjects with deficit schizophrenia (subjects with primary and persistent negative symptoms) as compared to subjects with non-deficit schizophrenia and healthy controls (HCs) (69, 89). One study (69) reported an association between N100 and primary and persistent negative symptoms. The authors of this study found that subjects with deficit schizophrenia, as compared to subjects with non-deficit schizophrenia and HCs, had a reduction in N100 amplitude for target tones and also topographic abnormalities for standard tones in brain areas involved in the evaluation of motivational relevance of events, auditory discrimination, and memory retrieval (69).

In contrast, the study conducted by Li et al. failed to find any specific associations between primary and persistent negative symptoms and N100, since both subjects with deficit and those with non-deficit schizophrenia presented the same alterations in N100 (reduced amplitude and delayed latency) as compared to HCs (89). Other two studies did not find a significant correlation between N100 and negative symptom severity (90, 91). While in a small sample of men with recent-onset psychosis, a correlation was found between N100 and negative symptom severity (85). In particular, the authors reported that men with recent onset psychosis had lower right-anterior N100, as compared to HCs, and that this abnormality correlated with the severity of negative symptoms, measured with the positive and negative syndrome scale (PANSS) (92). Starting from the assumption that the main generator of the anterior N100 is the anterior cingulate cortex, these results suggested that negative symptoms might be due to abnormalities in anterior cingulate cortex in modulating signal to noise ratio (85).

However, the majority of the above-mentioned studies (85, 89–91) used first generation rating scales, such as the PANSS (92) and the scale for the assessment of negative symptoms (SANS) (93) to assess negative symptoms. These assessment instruments present some limitations, as they include aspects that actually are not conceptualized as negative symptoms, but are mostly related to cognitive functions and disorganization (2). Furthermore, the study of Li et al. (89) used a proxy from the PANSS for categorizing subjects with deficit and non-deficit schizophrenia. However, it has been demonstrated that the proxy for categorizing DS and NDS patients has some problems in terms of face validity and temporal stability (2). The association between N100 abnormalities with the two negative symptom domains in SCZ has never been investigated.

Therefore, in the light of above observations, our study aims to fill the gap investigating in SCZ the relationships between N100 and the two negative symptom domains, evaluated with state-of-the-art instruments, in a large sample of SCZ.

To achieve this aim, the study investigated: (1) the differences in N100 parameters between subjects with SCZ and HCs; (2) the associations between N100 parameters with negative symptom domains in SCZ.

## Methods

### Study Participants

The study has been conducted as part of the add-on EEG study of the Italian Network for Research on Psychoses (3). One hundred and forty-eight SCZ and 70 HCs were recruited for the study, at five research sites in Naples, Foggia, Rome “Tor Vergata”, Rome “Sapienza”, and Salerno. The SCZ sample included individuals seen at the outpatient units of the five mentioned Italian university psychiatric clinics. All patients had a diagnosis of schizophrenia according to DSM-IV, confirmed with the Structured Clinical Interview for DSM IV-Patient version (SCID-I-P), and an age between 18 and 65 years.

The HCs sample was recruited from the community at the same sites mentioned above. Inclusion criteria for HCs were the absence of a current or lifetime Axis I or II psychiatric diagnosis. Exclusion criteria for both groups were: (a) a history of head trauma with loss of consciousness; (b) a history of moderate to severe mental retardation or of neurological diseases; (c) a history of alcohol and/or substance abuse in the last six months; (d) current pregnancy or lactation; and (e) inability to provide an informed consent. Schizophrenia with treatment modifications and/or hospitalization due to symptom exacerbation in the last three months were excluded.

The Ethics Committee of the involved institutions approved the electrophysiological add-on study. The study has been performed in accordance with the ethical standards laid down in the 1964 Declaration of Helsinki. All participants signed a written informed consent to participate after receiving a detailed explanation of the study procedures and goals.

### Clinical and Neurocognitive Assessments

All subjects recruited were evaluated for sociodemographic variables such as age, education, and gender, through a clinical form filled using every available source of information.

For SCZ, a semi-structured interview, the Brief Negative Symptom Scale (BNSS) was used to assess negative symptoms (94, 95). The scale includes 13 items, organized into six subscales (blunted affect, alogia, avolition, anhedonia, asociality, and a control subscale named distress). All the items are rated on a 7-point (0–6) scale, thus ranging from absent (0) to moderate (3) to extremely severe (6) symptoms (except distress for which the severity rating is reversed: 0 normal distress and 6 absent).

With regard to the two domains, the experiential domain was computed by summing the scores on the subscales anhedonia, avolition, and asociality; the expressive deficit was computed by summing the scores on the subscales blunted affect and alogia (94).

The PANSS was used to rate the severity of positive symptoms and disorganization (92). All items are rated on a 7-point scale from 1 to 7, ranging from absent (1) to moderate (4) to extremely severe (7). We also assessed depressive symptoms using the Calgary Depression Scale for Schizophrenia (CDSS) (96) and extrapyramidal symptoms using the St. Hans rating scale (SHRS) (97).

### EEG Recording Procedure

EEGs were recorded using two highly comparable EEG recording systems: EASYS2 (Brainscope, Prague) and Galileo MIZAR-sirius (EBNeuro, Florence). Before starting the study, a harmonization of the amplifier settings and recording procedure was carried out to ensure the same settings in all the centers. All EEGs were recorded using a cap electrode system with 29 unipolar leads (Fpz, Fz, Cz, Pz, Oz, F3, F4, C3, C4, FC5, FC6, P3, P4, O1, O2, Fp1, Fp2, F7, F8, T3, T4, T5, T6, AF3, AF4, PO7, PO8, right mastoid, and left mastoid), which were placed following the 10–20 system. All the leads were referenced to the linked earlobes (a resistor of 10 kΩ was interposed between the earlobe leads). A ground electrode was placed on the forehead.

For artifact monitoring, a horizontal electro-oculogram (hEOG) was recorded from the epicanthus of each eye, and a vertical EOG (vEOG) from the leads beneath and above the right eye. All impedances of the leads were kept below 5 kΩ. The EEG data were filtered with a band-pass of 0.15–70 Hz and recorded with a sampling rate of 512 Hz.

A calibration was performed for all channels, using a 50 μV sine wave, before each recording session. Subjects were seated in a reclining chair, in a sound attenuated room, minimizing eye movement or muscle tension. Subjects performed an auditory “odd-ball” task during which 320 standard stimuli (1,500 Hz, 80 dB) and 80 target stimuli, deviant for their frequency (1,000 Hz, 80 dB), were played. Patient were asked to press the button as fast as possible upon the appearance of every target stimulus. Participants who scored <60% on the behavioral target detection task were excluded from the analysis.

Participants were instructed not to drink coffee or tea and to abstain from smoking cigarettes in the 2 h before the beginning of the recording session and did not take psychotropic medications in the morning. Information on the quality of sleep during the night prior to the recording was collected and the EEG session was postponed if the subject reported a non-restoring sleep.

### EEG Data Preprocessing

One expert from the coordinating center (Naples) using Brain Vision Analyzer software (Brain Products, Munich, Germany) performed all the pre-processing analyses on data collected by the different recording sites. Data were parsed into epochs of 1,000 ms duration, which were time-locked to the onset of the cue and spanned from a 100 ms pre-stimulus period up to 900 ms post-stimulus. The recorded EEG was digitally filtered offline using a band-pass filter of 0.01–30 Hz. N100 waves were extracted in each subject by the averaging method in order to improve the signal/noise ratio, ruling out baseline activity not related to the stimulus. The N100 components for standard and target tones were analyzed separately. Trials with drifts larger than ±100 μV in any scalp electrode were rejected. If following artifacts and noisy trials removal, <40 usable target trials (50% of target trials) remained, the subject was excluded from the analysis. Data were baseline-corrected using the 100 ms time window preceding stimuli. N100 peaks were automatically marked using the “peak finder” function of Brain Analyzer, as the most negative peak point ranging from 80 to 120 ms post-stimulus. We analyzed amplitude and latency of N100 from the Fz, Cz, and Pz electrodes. Target stimuli also elicited a later auditory ERP known as the P3b, which is related to the allocation of attentive resources toward task relevant tones. Although the current study aimed to characterize the very early processing stages of auditory perception rather than higher-order processing phases, a control analysis was carried out to verify whether the later component was associated with negative symptoms. Findings concerning the difference between patients and controls for this component are reported elsewhere (Giordano et al., unpublished).

### Statistical Analysis

All statistical analyses were computed using SPSS Version 22.0 (IBM Corporation, 2014). Normality tests were performed on demographic, clinical, and electrophysiological variables to test distribution of data in order to set up parametric or non-parametric tests.

Mann-Whitney U-Tests and χ^2^-tests were used to compare SCZ and HCs on demographic characteristics. N100 amplitude and latency were entered separately into a two-factor repeated measures ANOVA design, incorporating electrode × stimulus type × group, with electrode and stimulus type as within subjects' variables and group as between subjects' factors. The Huynh-Feldt correction was applied. Significant main and interaction effects were further analyzed by *post-hoc* comparisons with Bonferroni adjusted alpha level using independent samples *t*-test and Mann-Whitney U-test.

Pearson or Spearman rank correlations, based on normality test results, were performed to test the relationships between N100 amplitude and latency for standard and target stimuli separately at the three midline electrodes (Fz, Cz, and Pz) with negative symptom severity (BNSS total score) in SCZ. For all the correlations considered, Bonferroni-Holm correction was applied in order to control for type-I error inflation, accordingly to the number of tests (three tests for each stimulus type, *p* < 0.016). Only when a significant correlation of BNSS total score with N100 measures was observed, correlations of the same measures with the two negative symptom domains (experiential domain and expressive deficit), and their component symptoms were further assessed (*p*-value threshold corrected accordingly to the number of symptom domains). Furthermore, if correlations with negative symptoms were statistically significant, we performed partial correlations to exclude the influence of positive and extrapyramidal symptoms, disorganization, and depression.

## Results

### Participants

One hundred and forty-eight SCZ and 70 HCs were originally enrolled as part of the add-on EEG study. However, 23 SCZ and 4 HCs did not complete the paradigm for the electrophysiological recording. Furthermore, 11 SCZ and 3 HCs were excluded either for the presence of artifacts in the ERP recordings or for poor behavioral performance on the active target recognition task. Thus, the final study sample consisted of 114 SCZ and 63 HCs.

### Demographic and Clinical Characteristics

Data on relevant demographic and clinic characteristics are provided in Table 1. The gender ratio was significantly different between the two groups (χ^2^ = 7.214; *p* < 0.01) since in the SCZ group the number of male subjects was higher, as compared to HCs; the mean age was not significantly different between the two sample groups (*U* = 2982.00; *p* > 0.05). Furthermore, as expected, SCZ had significantly lower education as compared to HCs (*U* = 2759.00; *p* < 0.01). Schizophrenia were characterized by mild to moderate severity of the negative symptoms (BNSS total score of 34.75) and absent to mild severity of both positive and disorganization dimensions (PANSS mean dimension score <9 for both). They had a low mean level of depression (CDSS total score <4) and of Parkinsonism (SHRS Parkinsonism score <1).

**Table 1:** Demographic characteristics and illness related variables.

	**SCZ (*n* = 114)**	**HCs (*n* = 63)**	**Statistics**	
**Gender**	81 M−33 W	32 M−31 W	χ^2^ = 7.214; ***p*** **= 0.007**	
	**Mean ± SD**	**Mean ± SD**	***t/U***	***p***
Age	36.86 ± 9.39	34.44 ± 12.48	*U* = 2982.00	0.062
Educational level (years)	12.35 ± 3.02	13.98 ± 4.04	*U* = 2759.00	**0.0083**
BNSS total score	34.75 ± 16.31			
BNSS expressive deficit domain	11.35 ± 7.27	–	–	–
BNSS experiential domain	21.11 ± 9.25	–	–	–
PANSS total	70.50 ± 19.41	–	–	–
PANSS negative factor	15.82 ± 5.84	–	–	–
PANSS positive factor	8.33 ± 4.74	–	–	–
PANSS disorganization Factor	8.60 ± 3.49	–	–	–
CDSS total score	3.24 ± 3.92	–	–	–
SHRS global Parkinsonism	0.86 ± 1.15	–	–	–

*BNSS, brief negative symptom scale; CDSS, the Calgary depression scale for schizophrenia; HCs, healthy controls; PANSS, positive and negative syndrome scale; SCZ, subjects with schizophrenia; SD, standard deviation; SHRS, the St. Hans rating scale for extrapyramidal syndrome. p-Values in bold indicate statistical significance*.

### Group Comparison on N100 Amplitude and Latency

Mean values of N100 amplitude (Table 2) and latency (Table 3) were calculated for SCZ and HCs.

**Table 2:** Comparisons of N100 mean amplitude for standard and target stimuli between subjects with schizophrenia and healthy controls.

**N100 amplitude**	**SCZ (*n* = 114)**	**HCs (*n* = 63)**	**Statistics**	
	**Mean ± SD**	**Mean ± SD**	***t/U***	***p***
Standard–Fz	−5.71 ± 2.94	−7.78 ± 3.22	*t* = 4.315	**<0.001**
Standard–Cz	−5.34 ± 2.79	−7.06 ± 2.91	*t* = 3.869	**<0.001**
Standard–Pz	−2.58 ± 2.01	−3.74 ± 1.87	*t* =3.752	**<0.001**
Target–Fz	−6.56 ± 3.18	−8.91 ± 3.68	*U* = 2165.00	**<0.001**
Target–Cz	−5.94 ± 3.34	−7.69 ± 3.45	*t* = 3.292	**<0.01**
Target–Pz	−2.55 ± 2.42	−4.04 ± 2.22	*U* = 2280.00	**<0.001**

*HCs, healthy controls; SCZ, subjects with schizophrenia; SD, standard deviation. p-Values in bold indicate statistical significance*.

**Table 3:** N100 mean latency for standard and target stimuli in subjects with schizophrenia and healthy controls.

**N100 latency**	**SCZ (*n* = 114)**	**HCs (*n* = 63)**
	**Mean ± SD**	**Mean ± SD**
Standard–Fz	88.55 ± 10.54	88.97 ± 9.46
Standard–Cz	87.90 ± 10.04	90.27 ± 11.29
Standard–Pz	88.38 ± 11.31	89.75 ± 12.84
Target–Fz	92.53 ± 12.23	92.26 ± 11.34
Target–Cz	90.66 ± 11.47	93.84 ± 11.93
Target–Pz	90.25 ± 11.95	90.37 ± 12.48

*HCs, healthy controls; SCZ, subjects with schizophrenia; SD, standard deviation. No comparisons between the two groups were made since the main effect of group was not significant (please refer to the text)*.

**Figure 1:**
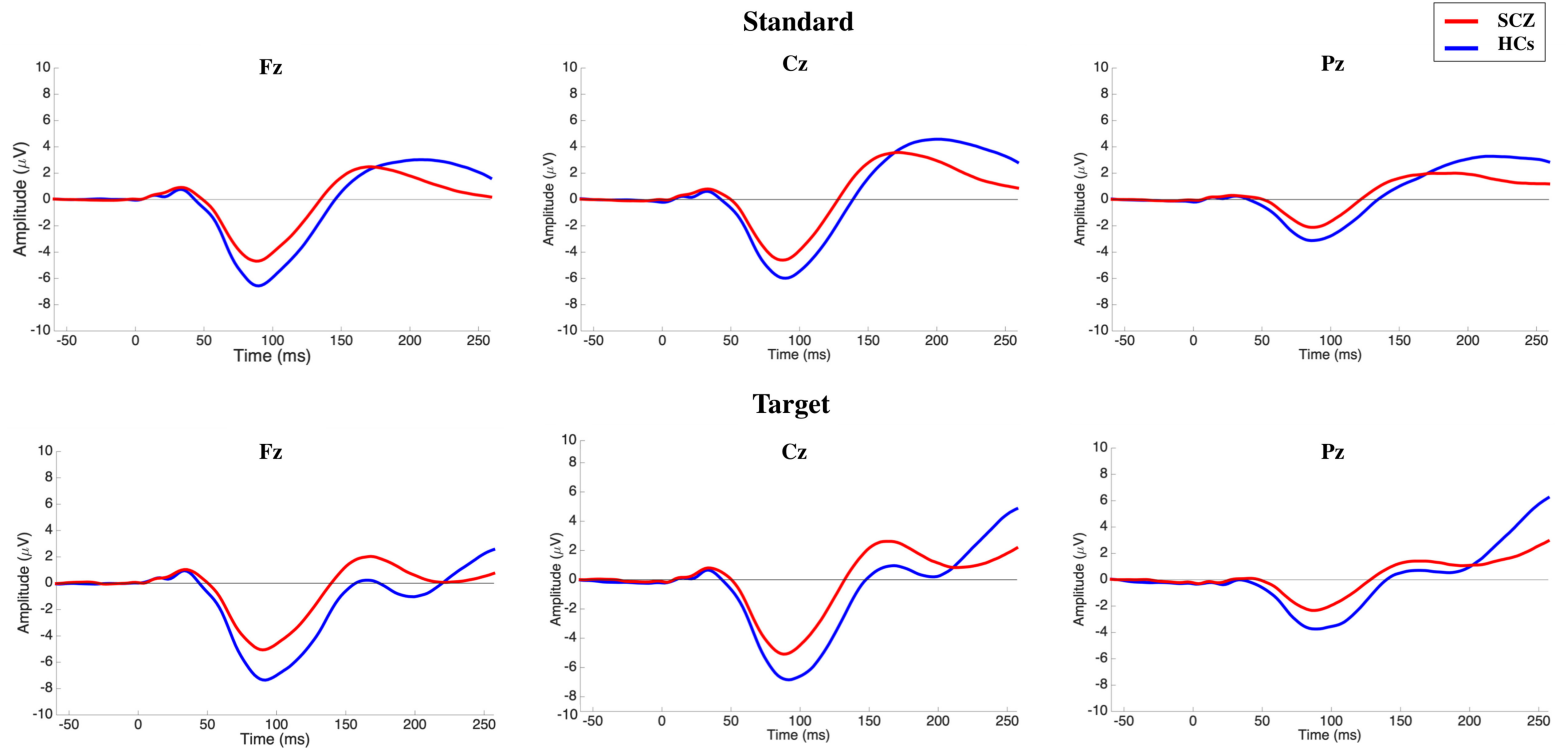
Grand-average N100 waves elicited by standard (top) and target (bottom) stimuli on the three midlines electrodes for subjects with schizophrenia (SCZ) and healthy controls (HCs). Following stimulus onset, the N100 component was measured in a time window within 80–120 ms. The potential was filtered between 0.01 and 30 Hz to optimize scoring of the N100 component.

No significant electrode × stimulus × group interaction [*F*_(1.724, 301.67)_ = 0.906; *p* > 0.05] on N100 amplitude was detected. A significant main effect of the electrode was recorded [*F*_(1.772, 310.18)_ = 354.03; *p* < 0.001; highest peaks amplitude recorded on Fz and Cz electrodes], while no significant electrode × group interaction was detected (*p* > 0.05). A significant main effect of the stimulus type was observed [*F*_(1, 175)_ = 27.658; *p* < 0.001; higher peak amplitude on target trials], but this was not influenced by group (*p* > 0.05). Finally, a main effect of group was found [*F*_(1, 175)_ = 20.272; *p* < 0.001]. Given the above group main effect and influence of stimulus type and electrode, the difference between the two groups were further investigated at each electrode level, separately for standard and target stimuli. *Post-hoc* analysis showed that remarkable reductions (*p* < 0.001) in N100 amplitude could be observed both in standard and target stimuli (lower N100 absolute value in SCZ; Figure 1; Table 2).

No significant interaction group × electrodes × stimulus type was detected for N100 latency [*F*_(1.757, 307.55)_ = 0.550; *p* > 0.05]. No significant electrode × group interaction or main effect of electrode was found (*p* > 0.05). A significant main effect was detected for stimulus type [*F*_(1, 175)_ = 15.976; *p* < 0.001; longer latency for target stimuli], which was not affected by the group variable (*p* > 0.05).

Finally, no significant main effect of group was detected [*F*_(1, 175)_ = 0. 542; *p* > 0.05]. Given the absence of a main effect of group, no *post-hoc* analysis for N100 latency was implemented (Table 3).

### Correlation Analysis Between N100 Characteristics and Negative Symptoms

**Figure 2:**
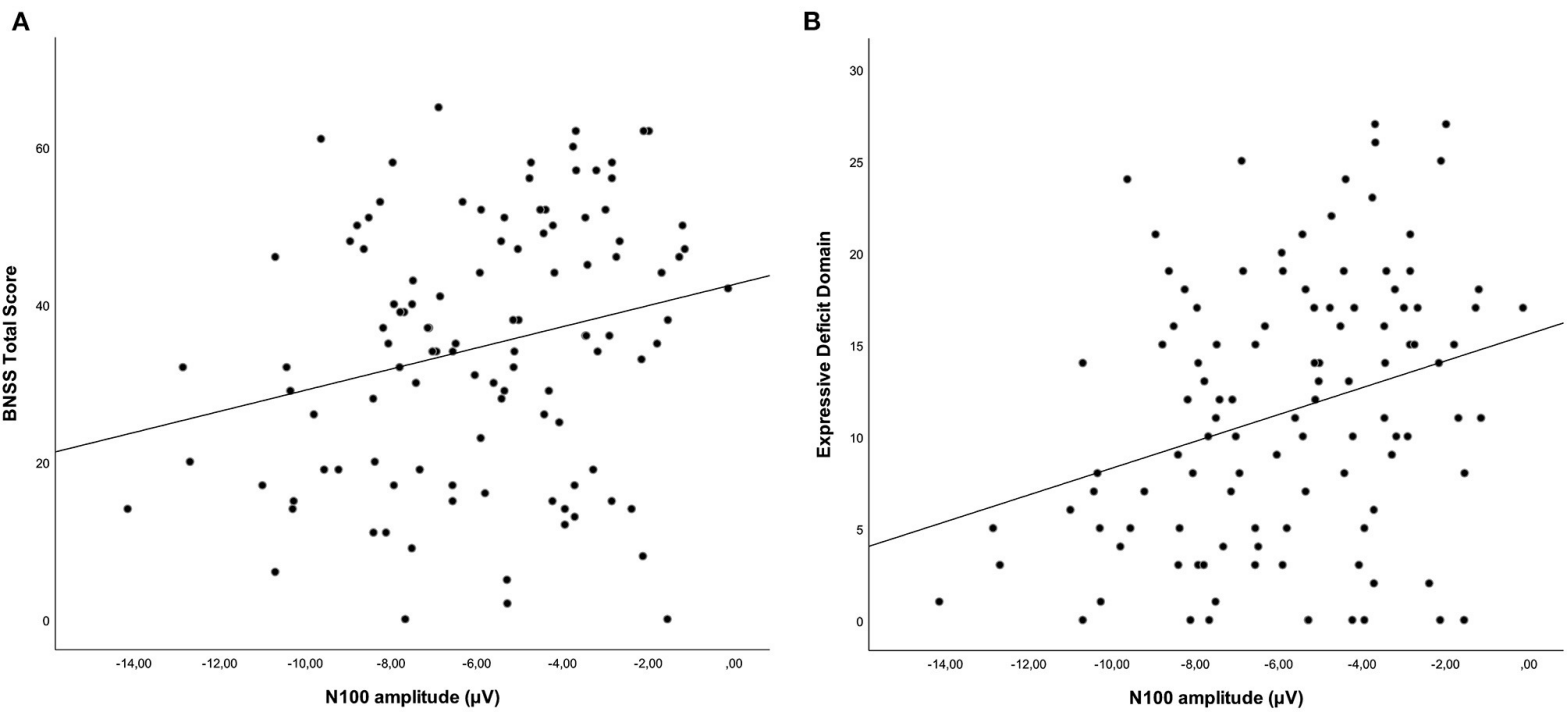
Correlations between standard-stimuli N100 amplitude (Fz electrode) with the BNSS total score **(A)** (*r*_*s*_ = 0.241; *p* = 0.011) and the expressive deficit domain **(B)** (*r*_*s*_ = 0.296; *p* = 0.002) in subjects with schizophrenia. Both correlations remained significant after controlling for the possible confounding effects of positive symptoms, extrapyramidal side effects, depression, and disorganization.

Correlations between N100 features and severity of negative symptoms, assessed through the BNSS total score, were initially performed. Correlations between BNSS total score and N100 amplitude and latency are reported in Tables 4, 5, respectively. We found that N100 amplitude recorded at Fz elicited by standard stimuli correlated with the BNSS total score (*r*_*s*_ = 0.241; *p* = 0.011) (Figure 2; Table 4). No significant associations between N100 latency and BNSS total score were observed (Table 5).

**Table 4:** Correlations between N100 amplitude for standard and target stimuli and BNSS total score in SCZ.

**N100 amplitude**	**BNSS total score**	
	**Spearman's correlation coefficient**	***p*-values**
Fz–Standard	0.241	**0.011**
Cz–Standard	0.089	0.351
Pz–Standard	0.132	0.167
Fz–Target	0.123	0.197
Cz–Target	0.030	0.754
Pz–Target	0.076	0.428

*BNSS, brief negative symptom scale. Significant p-value thresholds for the three correlations ran for each stimulus type (p < 0.016). p-values in bold indicate statistical significance*.

**Table 5:** Correlations between N100 latency for standard and target stimuli and BNSS total score in SCZ.

**N100 latency**	**BNSS total score**	
	**Spearman's correlation coefficient**	***p*-values**
Fz–Standard	0.052	0.586
Cz–Standard	0.094	0.328
Pz–Standard	0.113	0.236
Fz–Target	− 0.006	0.946
Cz–Target	−0.040	0.674
Pz–Target	− 0.015	0.878

*BNSS, brief negative symptom scale; Significant p-value thresholds for the three correlations ran for each stimulus type (p < 0.016)*.

Furthermore, when we considered the two domains of negative symptoms, we found a different pattern of correlations between these domains and N100 amplitude. In particular, while a correlation was observed between N100 amplitude (standard stimuli–Fz electrode) and the expressive deficit domain (*r*_*s*_ = 0.296; *p* = 0.002) (Figure 2; Table 6), no significant correlation was found for the experiential domain (*r*_*s*_ = 0.188; *p* = 0.051) (Table 6). Since the *p*-value of this last correlation was close to the threshold value, we performed an exploratory analysis focusing on the correlations between N100 amplitude and all the symptoms constituting the experiential domain (avolition, anhedonia, and asociality). The correlations between the N100 amplitude and avolition (*r*_*s*_ = 0.075; *p* = 0.445) and asociality (*r*_*s*_ = 0.040; *p* = 0.686) were not statistically significant, while the correlation with anhedonia did not survive correction for multiple tests (*r*_*s*_ = 0.205; *p* = 0.035).

**Table 6:** Correlations between N100 amplitude (standard stimuli–Fz electrode) and negative symptom domains.

**BNSS**	**N100 amplitude (standard stimuli–Fz electrode)**	
	**Spearman's correlation coefficient**	***p*-values**
Experiential domain	0.188	0.051
Expressive deficit	0.296	0.002^*^
Blunted affect	0.240	0.011^*^
Alogia	0.253	0.007^*^

*BNSS, brief negative symptom scale. Significant p-value thresholds for the three correlations ran for each stimulus type (p <0.025). p-values in bold indicate statistical significance*.

* *The correlation remained significant when controlling for positive and extrapyramidal symptoms, disorganization and depression*.

Finally, within the expressive deficit, both blunted affect (*r*_*s*_ = 0.240; *p* = 0.011) and alogia (*r*_*s*_ = 0.253 *p* = 0.007) had the same pattern of correlation with N100 (Table 6). All correlations remained significant after controlling for the possible confounding effects of positive symptoms, extrapyramidal side effects, depression, and disorganization.

### Control Analysis of Correlations of P3b With Negative Symptoms

No association of P3b with negative symptoms was observed in the study. In particular, no significant correlations were found between P3b amplitude and the BNSS total (*r*_*s*_ = −0.054; *p* = 0.575) or the experiential (*r*_*s*_ = −0.053; *p* = 0.577) and the expressive deficit (*r*_*s*_ = −0.060; *p* = 0.533) domains. Finally, no significant correlations were found between P3b latency and the BNSS total (*r*_*s*_ = −0.046; *p* = 0.635) and the experiential (*r*_*s*_ = −0.037; *p* = 0.701) and the expressive deficit (*r*_*s*_ = −0.083; *p* = 0.387) domains.

## Discussion

The current study aimed to investigate auditory-elicited N100 in SCZ and its association with negative symptom domains. The two main aims were: (1) to identify differences in N100 amplitude between SCZ and HCs; (2) to investigate the presence of associations between N100 and negative symptom domains (experiential and expressive deficit) in SCZ. The main results of our study included: (1) N100 amplitude was reduced in SCZ, compared to HCs, while no significant differences were detected in N100 latency between the two groups; (2) negative symptoms, assessed by BNSS scale, showed an association with N100 amplitude for standard stimuli; (3) expressive deficit, but not the experiential domain, was associated with N100 amplitude; and (4) both blunted affect and alogia were associated with N100 amplitude.

N100 amplitude was reduced in SCZ compared to HCs, for both standard and target stimuli. These results are in line with previous literature findings, which robustly documented diminished N100 amplitude in SCZ (67–71, 73, 76, 77). Abnormalities of N100 are already detectable in early stages of the disease and in high-risk individuals (74, 75) and, therefore, have been proposed as indicators of brain functional changes related to schizophrenia vulnerability (98). In line with this hypothesis, N100 amplitude deficit has also been recorded in unaffected first-degree relatives of subjects with SCZ (72). Using topographic analysis, such as low-resolution electromagnetic tomography analysis (LORETA), it is possible to detect the main brain areas involved in N100 generation: the primary auditory cortex, the dorsolateral prefrontal cortex, and the anterior cingulate (69, 99). Abnormalities in these areas, along with widespread connectivity alterations are consistently reported in neuroimaging studies conducted in SCZ (8, 100, 101).

The N100 is regarded as an index of early visual and auditory processing, which is also influenced by selective attention and unpredictability of the stimuli. Therefore, the reduction in N100 amplitude in SCZ is interpreted as a deficit in early sensory processing of the stimulus, an aspect well documented in schizophrenia both through behavioral and neurophysiology studies, since the earliest stages of the disease (67, 70, 75, 79, 85). Deficits in early visual and auditory processing, along with aberrations in the integration of simultaneous and multisensory stimulation, might lead to impairment also in higher-level functions (86, 102–105).

The second part of our study aimed to evaluate the relationship between N100 and negative symptoms. Previous studies have found an association between dysfunctions in N100 elicitation in SCZ and auditory hallucinations (80–82), antipsychotic intake (67), attention deficits (83, 84), and negative symptoms (69, 85).

As reported in the Introduction, the association between N100 abnormalities and negative symptoms remains unclear since results reported by different studies are inconsistent (69, 85, 89–91). However, the majority of the above-mentioned studies (85, 89–91) used first generation rating scales, such as the PANSS (92) and the SANS (93) to assess negative symptoms. These assessment instruments present some limitations, as they include aspects that actually are not conceptualized as negative symptoms, but are mostly related to cognitive functions and disorganization (2). In addition, previous studies did not investigate associations between N100 and the two negative symptom domains.

In a large sample of stabilized subjects with chronic schizophrenia, our study demonstrated a relationship between N100 abnormalities with negative symptoms. The strength of this finding stem from fact that negative symptoms were evaluated with the BNSS, a second-generation rating scale in line with the current conceptualization of negative symptoms, and that as documented by partial correlation analysis, this outcome was not mediated by positive symptoms, extrapyramidal side effects, disorganization, or depression, frequently causing secondary negative symptoms within negative symptoms, the expressive deficit domain was strongly correlated with N100 amplitude, as compared to the experiential domain. In particular, although the *p*-value of association between the experiential domain and the N100 was close to threshold, none of the symptoms belonging to this domain was significantly correlated with N100 amplitude, while the expressive deficit domain and its subcomponent symptoms were correlated with N100.

The presence of an association of N100 amplitude with only one of the two negative symptom domains is in agreement with previous results that suggest the existence of separate neurobiological mechanisms at the core of the experiential domain and expressive deficit (8, 10, 15, 16, 37, 58).

Indeed, neuroimaging studies have provided a rich evidence of the possible faulty neuronal circuits underlying the two domains of negative symptoms. The experiential domain seems to be related to abnormalities in brain networks regulating different aspects of motivation, and probably to impairment in executive functions. On the other side, the pathophysiological mechanisms at the basis of the expressive deficit domain remain less understood (8, 10, 15, 16). This domain of negative symptoms has been related to deficit in neurocognitive skills, social cognition abilities, and neurological soft signs, which comprise also subtle deficits in sensory integration, along with motor coordination, and sequencing of complex motor acts (8). These associations seem to pinpoint that this domain is related to a diffuse neurodevelopmental disconnectivity.

According to the hypothesis of the limited cognitive resource, expressive deficit symptoms, in particular alogia, might depend on deficits in different cognitive functions, such as semantic memory organization and verbal fluency. Starting from limited cognitive resources, in “high-load” situations (e.g., social situations) subjects are exposed to high cognitive demands and might allocate less cognitive resources to speech production (15). As we have reported above, reduced N100 amplitude is an index of deficits in sensory processing and sensory gating, a well-replicated finding in SCZ. It has been proposed that alterations in sensory gating of N100 cause sensory flooding and defective processing of information to the brain, contributing to the symptoms of SCZ (91). Given the relationship between N100 and sensory processing deficits, our study demonstrated a connection between deficits in sensory processing with negative symptom severity, in particular those belonging to the expressive deficit. A possible interpretation of this connection is based on some “cascade” models that have hypothesized that impairment in early sensory processing might contribute to deficits in higher-level processing which are related to negative symptoms, leading to poor functioning (86–88, 106).

Certain limitations of this study should be taken into account. For instance, age and pharmacological treatment might have had an impact on our results. In our study, we used a sample in which subjects were matched for age: therefore, we could exclude the effect of age on the differences between HCs and SCZ. With regard to medication, we excluded the confounding effect of medication on correlation between N100 and negative symptoms, using partial correlation analysis in which we controlled for extrapyramidal symptoms that might cause secondary negative symptoms. However, further studies including drug-naïve subjects at their first episode, as well as subjects at high risk for psychosis, using a proper characterization of negative symptoms, are needed in order to disentangle different neurobiological underpinnings of negative symptom domains.

## Conclusions

In conclusion, in line with previous studies, our results suggested that chronic individuals with schizophrenia are affected by neurophysiological abnormalities in early stages of auditory processing, as indexed by reduced N100 amplitude. In addition, we reported a correlation between reductions of N100 amplitude and severity of the expressive deficit domain, while no correlation was found with the experiential domain. These results reinforce the hypothesis of separate neurophysiological correlates of the two negative symptom domains. Furthermore, previous models have hypothesized a concatenation of pathological features starting from impairment in early sensory processing up to deficits in higher-level processing that could lead to negative symptoms, and finally might contribute to poor functioning in real life. Further studies, including large sample sizes, a proper characterization of negative symptoms, and an analysis of pathways to functional outcome, are needed.

Improving knowledge in the pathophysiology of different aspects of negative symptoms and their relative contribution to poor functioning is one of the main goals of the research, since it could help in the design and implementation of effective treatments for negative symptoms, which unfortunately still represent an unmet need in the care of SCZ.

## Italian Network for Research on Psychoses

Members of the Italian Network for Research on Psychoses participating in the add-on EEG study include: Eleonora Merlotti and Giuseppe Piegari, University of Campania “Luigi Vanvitelli”; Girolamo Francavilla and Flavia A. Padalino, University of Foggia; Cinzia Niolu and Michele Ribolsi, University of Rome Tor Vergata; Roberto Brugnoli and Paolo Girardi, University of Rome “La Sapienza”; Giulio Corrivetti and Francesca Marciello, University of Salerno.

## Data Availability Statement

The original contributions presented in the study are included in the article/supplementary material, further inquiries can be directed to the corresponding author/s.

## Ethics Statement

The studies involving human participants were reviewed and approved by Comitato Etico Università degli Studi della Campania Luigi Vanvitelli—A.O.U. Luigi Vanvitelli and A.O.R.N. Ospedali dei Colli. The patients/participants provided their written informed consent to participate in this study.

## Author Contributions

The project idea was initiated by SG, involving a collaboration with GMG, FB, AP, GDL, and MM. SG and GMG planned the experimental procedures. GMG, FB, and AP performed the analyzes of the data and wrote the first draft of the manuscript. All authors were responsible for the interpretation of the analyzes, contributed to critically revising the content, and approved the final manuscript for submission to Frontiers in Psychiatry.

## Funding

This study was funded by the Italian Ministry of Education (grant number: 2010XP2XR4), the Italian Society of Psychopathology (SOPSI), the Italian Society of Biological Psychiatry (SIPB), Roche, Switzerland; Lilly, United States; AstraZeneca, United Kingdom; Lundbeck Foundation, Denmark; and Bristol-Myers Squibb, United Kingdom. These entities had no role in the study design; in the collection, analysis and interpretation of data; in the writing of the report and in the decision to submit the paper for publication.

## Conflict of Interest

The authors declare that the research was conducted in the absence of any commercial or financial relationships that could be construed as a potential conflict of interest.

## Publisher's Note

All claims expressed in this article are solely those of the authors and do not necessarily represent those of their affiliated organizations, or those of the publisher, the editors and the reviewers. Any product that may be evaluated in this article, or claim that may be made by its manufacturer, is not guaranteed or endorsed by the publisher.
